# Two-layer spatial frequency domain imaging of compression-induced hemodynamic changes in breast tissue

**DOI:** 10.1117/1.JBO.26.5.056005

**Published:** 2021-05-24

**Authors:** Constance M. Robbins, Syeda Tabassum, Molly F. Baumhauer, Jason Yang, James F. Antaki, Jana M. Kainerstorfer

**Affiliations:** a Carnegie Mellon University, Department of Biomedical Engineering, Pittsburgh, Pennsylvania, United States; b Carnegie Mellon University, Department of Physics, Pittsburgh, Pennsylvania, United States; c Cornell University, School of Biomedical Engineering, Ithaca, New York, United States; d Carnegie Mellon University, Neuroscience Institute, Pittsburgh, Pennsylvania, United States

**Keywords:** spatial frequency domain imaging, diffuse optics, breast imaging, tissue optics, hemodynamics

## Abstract

**Significance**: Longitudinal tracking of hemodynamic changes in the breast has shown potential for neoadjuvant chemotherapy (NAC) outcome prediction. Spatial frequency domain imaging (SFDI) could be suitable for frequent monitoring of shallow breast tumors, but strong sensitivity to superficial absorbers presents a challenge.

**Aim**: We investigated the efficacy of a two-layer SFDI inverse model that accounts for varying melanin concentration in the skin to improve discrimination of optical properties of deep tissue of the breast.

**Approach**: Hemodynamic changes in response to localized breast compression were measured in 13 healthy volunteers using a handheld SFDI device. Epidermis optical thickness was determined based on spectral fitting of the model output and used to calculate subcutaneous optical properties.

**Results**: Optical properties from a homogeneous model yielded physiologically unreasonable absorption and scattering coefficients for highly pigmented volunteers. The two-layer model compensated for the effect of melanin and yielded properties in the expected range for healthy breast. Extracted epidermal optical thickness was higher for higher Fitzpatrick types. Compression induced a decrease in total hemoglobin consistent with tissue blanching.

**Conclusions**: The handheld SFDI device and two-layer model show potential for imaging hemodynamic responses that potentially could help predict efficacy of NAC in patients of varying skin tones.

## 1 Introduction

Near-infrared (NIR) optical imaging methods have shown potential for prediction of treatment outcome in women undergoing neoadjuvant chemotherapy (NAC) for breast cancer via longitudinal tracking of hemoglobin concentration within the breast.^1^^–^^13^ Pathologic complete response (pCR) to NAC (assessed histologically after surgery) is associated with improved survival,^14^^,^^15^ but the results of structural imaging modalities such as ultrasonography and x-ray mammography are poor predictors of patient outcome.^16^^,^^17^ Predicating the response early in the course of therapy could potentially enable treatment alternatives. NIR optical methods are attractive for this application for their non-invasiveness and sensitivity to endogenous contrast from hemoglobin, which is related to the vascularization and function of the tumor. Changes in tumor vasculature and tissue metabolism have been shown to occur earlier in therapy than changes in tumor size and shape.^1^^,^^6^ In particular, an oxyhemoglobin flare associated with treatment response has been observed as soon as one day after the start of therapy in some NAC regimens, possibly arising from increased perfusion due to an acute inflammatory response.^5^^,^^8^ The multi-center ACRIN 6691 trial of diffuse optical spectroscopic imaging (DOSI) found tumor tissue oxygen saturation measured within 10 days of treatment to be predictive of pCR.^12^ An independent study of diffuse optical tomography (DOT) for NAC monitoring found that a decrease in total hemoglobin (tHb) concentration at the two-week time point correlated with residual cancer burden.^7^ Therefore, we hypothesize that an inexpensive bedside-compatible system suitable for frequent monitoring could provide valuable insight into these hemodynamic changes that occur quickly after the initiation of NAC.

In addition to quantifying baseline hemoglobin concentrations, hemodynamic changes to a perturbation in the tissue offers another modality for tracking the effect of NAC on breast tumors. Breast tumor vasculature tends to be disorganized and exhibit abnormal flow,^18^ leading to differential hemodynamic responses to perturbations, such as breath-holding and mechanical compression, compared with healthy breast. Flexman et al.^19^ reported that breast tumors exhibit slower return to baseline after a breath-holding maneuver compared with healthy breast. In response to breast compression, Carp et al.^20^ reported a faster decrease in tissue oxygen saturation of hemoglobin for breast tumors compared with healthy tissue, and a slower reperfusion of the tumor region after the release of compression pressure. In both studies, these differential hemodynamics were shown to normalize during NAC in responding patients.^3^^,^^4^

Due to the significant cost and large footprint of DOT instrumentation, various less expensive and more portable approaches have also been developed. The DOSI technology consists of combined frequency domain and broadband continuous-wave NIRS and utilizes a handheld probe that is moved across the breast to create a 2D image. However, the need to acquire multiple point measurements prevents imaging of hemodynamic responses to perturbations. Portable approaches to dynamic breast imaging include the P-scan handheld continuous-wave NIRS device^21^^,^^22^ used to record hemodynamic response to breast compression, and more recently, a flexible wearable NIRS probe^23^^,^^24^ has been developed that conforms to the surface of the breast to capture hemodynamics with temporal and spatial resolution.

We have chosen to pursue a handheld breast imaging method based on spatial frequency domain imaging (SFDI). Because SFDI can be built inexpensively at small form-factor, we envision this device to be suitable for frequent measurement during therapy, including in the patient’s home. SFDI is a wide-field diffuse optical imaging method capable of quantifying absorption and reduced scattering coefficients. Reflectance of spatially modulated illumination is used to characterize the spatial frequency dependent attenuation of light by the tissue, from which optical properties can be obtained by fitting to a theoretical model of light propagation within the tissue. In the simplest implementations, this light propagation model includes the assumption of tissue homogeneity, based either on the standard diffusion approximation^25^ or on single-layer Monte Carlo (MC) simulations. Though more spatial frequencies can be used, a minimum of two is sufficient to quantify the optical properties of a homogeneous medium, with 0 and $0.1\;{\mathrm{mm}}^{-1}$ being one frequency pair that provides good separation of absorption, $\mu_{a}$, and reduced scattering, ${\mu_{s}}^{\prime }$, coefficients over physiologically relevant ranges.^26^^,^^27^

A known drawback of SFDI is limited penetration depth, on the order of <1  cm, and therefore high sensitivity to superficial tissue.^28^^,^^29^ We have shown recently that this limitation can partially be overcome by the use of localized tissue compression, for instance to bring a stiff lesion within the sensitivity range of the imaging method.^30^ However, interference from superficial absorbers, such as melanin in the epidermis, presents a significant challenge to accurate reconstruction of subcutaneous optical properties.

Various approaches have been developed to account for the presence of skin pigmentation. Spatial frequency domain spectroscopy (SFDS) utilizes broadband spatially modulated illumination detected by fiber coupled spectrometer rather than a camera, trading spatial information for spectral resolution. SFDS leverages the differential penetration depth of the visible and NIR wavelength ranges to provide estimates of layer-specific chromophore concentrations^31^ and has been used to separate melanin concentration in the skin from underlying hemodynamics during venous occlusion.^32^ However, acquisition times of 10 to 30 s are required, precluding capture of faster hemodynamic changes. Additionally, the wider field of view and spatial resolution granted by SFDI would be advantageous for the application of tumor monitoring, as it would enable segmentation of the tumor from the healthy background. For example, Yudovsky et al.^33^ described a neural network model for analysis of 2D SFDI images that can decouple epidermis optical depth from underlying $\mu_{a}$ and ${\mu_{s}}^{\prime }$. The method requires the projection of six spatial frequencies and detection of eight wavelengths with a tunable filter, at considerably higher cost than detection with CMOS or CCD camera.

A different approach introduced by Tabassum et al.^34^ utilizes a two-layer SFDI inverse model consisting of a lookup table (LUT) derived from layered rather than homogeneous Monte Carlo simulations. Optical properties and superficial layer thickness are fixed. Their approach demonstrated improved ability to recover bulk layer optical properties compared to a homogeneous model. Though this model requires prior knowledge of skin layer properties, it offers the advantage of requiring only two spatial frequencies and can utilize any number of wavelengths, along with the wealth of spatial information provided by detection by camera. However, the practical clinical utility of this model is limited due to the wide variation of skin absorption properties between individuals. To address this limitation, we developed a three-dimensional (3D) MC LUT in which $\mu_{a}$ of the top layer can be varied, representing a wide range of human skin tones. An optimization procedure for extraction of the top layer $\mu_{a}$ within the LUT based on spectral fitting of the bulk optical properties to hemoglobin concentrations is presented.

## 2 Methods

An SFDI-based handheld breast imaging device was used to record hemodynamic response to localized breast compression in 13 healthy subjects of varying skin tone. A two-layer inverse model was employed in which the product of top layer (epidermis) absorption coefficient and epidermal thickness was estimated based on goodness of fit of calculated subcutaneous absorption with hemoglobin extinction coefficients.

### 2.1 SFDI: Theory and Instrumentation

We previously developed a handheld SFDI device for breast imaging, described previously.^35^ Briefly, the device consists of a monochrome CMOS camera (Texas instruments, Dallas, Texas) and digital light projector (LightCrafter, Texas instruments, Dallas, Texas) enclosed in acrylic housing. The original RGB LEDs of the projector were replaced with three high power surface-mount LEDs with peak emission wavelengths, λ, of 662, 735, and 859 nm (Luminus Devices, Sunnyvale, California). The two dichroic mirrors in the projector were replaced with ones with cutoff wavelength of 697 and 801 nm (FF697-SDi01 and FF801-Di02, Semrock optics, Rochester, New York). The original collimation lenses in front of the LEDs were also replaced (49874, Edmund Optics, Barrington, New Jersey). Crossed polarizers (LPVIS050 and LPNIRE100-B, Thorlabs, Newton, New Jersey) were added to reduce specular reflection. A glass optical window of diameter 50 mm (WG12012-B, Thorlabs, Newton, New Jersey) was used to make contact with the skin and four bar-style load cells were used to record the normal force applied to the tissue (Phidgets, Calgary, Alberta, Canada). Pattern projection and image acquisition from the camera was performed automatically by a custom MATLAB application implemented on a laptop computer. Real-time feedback of force applied was displayed on the computer screen in the form of a line graph. This was monitored by the operator to aid in applying a consistent pressure during the compression period.

Sinusoidal illumination at spatial frequency $f_{x}=0.1\;{\mathrm{mm}}^{-1}$ was projected at three equally spaced phase shifts for each wavelength, λ. The nine patterns (three phases, three wavelengths) were projected sequentially, with three blank frames included in the sequence to serve as a marker to identify the start of the sequence. Projection of the patterns was synchronized to the camera exposure with a frame rate of 40 fps. Each set of 12 frames, representing one multi-wavelength SFDI measurement, required a total of 300 ms for acquisition. One SFDI measurement was captured every 2 s. Following image acquisition, image demodulation was performed in real time. The zero frequency magnitude, M(0), and the magnitude at $f_{x}=0.1\;{\mathrm{mm}}^{-1}$, M(0.1), were calculated on a pixel-by-pixel basis according to Eqs. (1) and (2):^28^

$$M(0)=(I_{1}+I_{2}+I_{3})/3,$$ (1)

$$M(0.1)=\frac{\sqrt{2}}{3}\cdot \sqrt{{\left(I_{1}-I_{2}\right)}^{2}+{\left(I_{2}-I_{3}\right)}^{2}+{\left(I_{3}-I_{2}\right)}^{2}},$$ (2)

where $I_{1}$, $I_{2}$, and $I_{3}$ are the measured intensities of the three phases of sinusoidal illumination.

Calibration images were taken from a polydimethylsiloxane reference phantom of known optical properties, to obtain $M_{ref}(f_{x})$. The reference phantom was assessed by a commercial frequency domain near-infrared spectroscopy system (OxiplexTS, ISS, Champaign, Illinois) and found to have $\mu_{a}$ of 0.047 and $0.052\;{\mathrm{cm}}^{-1}$ (at 690 and 830 nm, respectively) and $\mu_{s}^{\prime }$ of 7.1 and $5.3\;{\mathrm{cm}}^{-1}$. To estimate optical properties at the three λ of interest (662, 735, and 859 nm), $\mu_{s}^{\prime }(\lambda )$ was assumed to follow a power law decay function as provided in Eq. (3)^2^

$$\mu_{s}^{\prime }(\lambda )=A{\left(\lambda /\lambda_{0}\right)}^{-b},$$ (3)

and $\mu_{a}$ was assumed to not vary significantly with λ and the average of the measured values was used for all λ. Using these known optical properties, the theoretical diffuse reflectance of the reference phantom, $R_{d,ref}(f_{x})$, was calculated using a Monte Carlo forward LUT for a homogeneous medium. Diffuse reflectance of the measured tissue, $R_{d,}(f_{x})$, was calculated according to

$$R_{d}(f_{x})=\frac{M(f_{x})}{M_{ref}(f_{x})}\cdot R_{d,ref}(f_{x}).$$ (4)

Conventionally, $R_{d}(f_{x})$ at two $f_{x}$ is inputted to a two-$f_{x}$ LUT to extract tissue $\mu_{a}$ and $\mu_{s}^{\prime }$. Details of optical properties optimization and extraction specific to the two-layer model are provided in Sec. 2.3. The processing steps were repeated for each of the three λ to generate wavelength-dependent values of $\mu_{a}(\lambda )$ and $\mu_{s}^{\prime }(\lambda )$. Using extracted values of $\mu_{a}(\lambda )$, concentration of oxy-, HbO (μM), deoxy-, Hb (μM), and tHb (μM), and oxygen saturation of hemoglobin, ${\mathrm{StO}}_{2}=\mathrm{HbO}/\mathrm{tHb}$, were calculated. Scattering amplitude, A, and scattering power, b, were also computed by fitting of $\mu_{s}^{\prime }(\lambda )$ using Eq. 3, where the reference wavelength, $\lambda_{0}$, was chosen to be 735 nm.

### 2.2 Human Subjects Imaging Protocol

Human subject testing was approved by the Carnegie Mellon Institutional Review Board under protocol STUDY2015_00000046. Thirteen healthy volunteers were recruited through the Pitt+Me online portal.^36^ Six returned for a second imaging session, for a total of 19 imaging sessions. All subjects were female and ranged in age from 22 to 61 years with no history of diabetes or vascular disease. Enrolled subjects comprised a range of skin tones, as detailed below. Informed consent was obtained from all subjects at the beginning of each visit. A Fitzpatrick skin score was then assessed via a questionnaire adapted from Eilers et al.^37^ The two questions were posed verbally to the subject and the experimenter would record her answers and assign a Fitzpatrick score based on the criteria described in Eilers et al.^37^ The questions were “If after several months of not being in the sun, you stayed outdoors for about 1 hour at noon for the first time in the summer without sunscreen, what would happen to your skin? Would it become pink/red, irritated, tender, or itchy?” and “Over the next 7 days, would you develop a tan or notice your skin becoming darker?” The resulting Fitzpatrick scores of the study population are provided in Table 1. Fitzpatrick score of I corresponds to the lightest skin tone and VI to the darkest. Fitzpatrick score was unavailable for two subjects, which were estimated to be type III or lower.

**Table 1:** Distribution of Fitzpatrick skin scale scores for 11 of the 13 study participants. The remaining two participants are estimated to be type III or lower.

	I	II	III	IV	V	VI
# of subjects	1	0	3	1	3	3

**Fig. 1:**
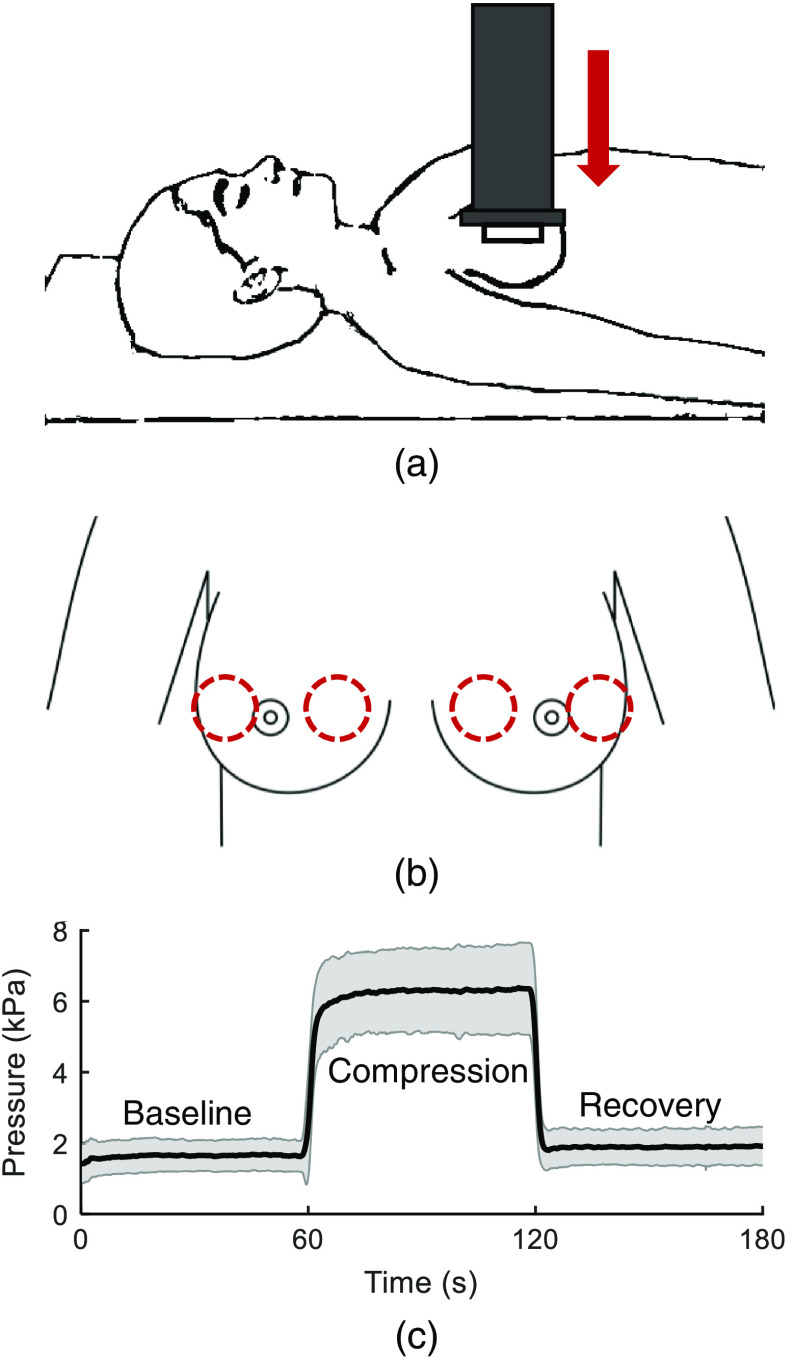
(a) Subject positioning and direction of pressure applied with handheld imager. (b) Approximate location of two imaging locations on each breast. Image adapted from: Ref. 38 (c) average pressure applied over all 3-min measurements, shaded region indicates standard deviation (n=71).

The experimental setup is shown in Fig. 1. The handheld device was used to apply local compression to the breast by pressing down against the ribcage at various locations on the breast. Subjects were lying in supine position while pressure was applied with the handheld device at two locations: one lateral to the nipple and one medial to the nipple. Five measurements were unusable due to technical failure of the imaging device, resulting in 71 measurements total. Each measurement consisted of 1 min of baseline measurement while the device was in contact with skin with minimal pressure, then one minute of sustained target pressure of 7.6 kPa, followed by a return to baseline pressure for one minute of recovery [Fig. 1(c)]. Petroleum jelly was used between the glass and the skin to ensure even contact and reduce trapped air. Over all measurements, an average pressure of 6.40  kPa±0.85  kPa was reached during compression.

### 2.3 Signal Processing

#### 2.3.1 Two-layered modeling of human breast

To simulate human skin, a two-layer model was developed in which the top layer mimicked the non-perfused epidermis, and the bottom layer represented the subcutaneous tissue. The chromophores contributing to absorption are melanin in the epidermis layer, and HbO and Hb in the subcutaneous layer. The thickness and scattering coefficient, $\mu_{s,epi}^{\prime }(\lambda )$, in the epidermis layer were held constant. The epidermis layer thickness was fixed at 110  μm (within the range of reported human epidermal thickness^33^^,^^39^^,^^40^), and $\mu_{s,epi}^{\prime }(\lambda )$ was set at 22.16, 16.89, and $11.62\;{\mathrm{cm}}^{-1}$ for 662, 735, and 859 nm, respectively.^41^ The absorption coefficient of the epidermis layer, $\mu_{a,epi}(\lambda )$, was determined empirically, as described in Sec. 2.3.3. The thickness of the subcutaneous layer was fixed at 10 cm and subcutaneous optical properties (output parameters of the model) were defined within the range of 0.01 to $0.75\;{\mathrm{cm}}^{-1}$ for $\mu_{a,sub}(\lambda )$, and 4 to $20\;{\mathrm{cm}}^{-1}$ for $\mu_{s,sub}^{\prime }(\lambda )$. As the thickness of the subcutaneous layer was approximately 40 times the maximum transport mean free path, $l^{*}=1/(\mu_{a}+\mu_{s}^{\prime })$, this layer mimicked a semi-infinite medium. After optimization of the participant-specific $\mu_{a,epi}(\lambda )$, the two-layer model was used to produce $\mu_{a,sub}(\lambda )$ and $\mu_{s,sub}^{\prime }(\lambda )$.

#### 2.3.2 Generation of two-layer Monte Carlo Lookup Table

Optical properties from SFDI were extracted based on a two-layer look up table, which was created using MC simulations using the virtual tissue simulator (VTS) software (Beckman Laser Institute, University of California, Irvine, California). VTS utilizes the method described by Gardner and Venugopalan^42^ to compute diffuse reflectance, $R_{d}$, natively in the spatial frequency domain. For all layers in all the simulations, anisotropy was prescribed as g=0.8, refractive index as n=1.4, and $1\times 10^{6}\text{ photons}$ were launched for each simulation. MC simulations were performed for the three wavelengths, therefore three $\mu_{s,epi}^{\prime }$ (22.16, 16.89, and $11.62\;{\mathrm{cm}}^{-1}$) and 17 increments of $\mu_{s,sub}^{\prime }$ ranging from 4 to $20\;{\mathrm{cm}}^{-1}$. In initial simulations, a constant $\mu_{a}$ of $0.06\;{\mathrm{cm}}^{-1}$ was used for both layers. Later in post processing, absorption coefficients were scaled to the desired values separately for both layers using Beers law. Here, $\mu_{a,epi}$ was varied between 0 and $20\;{\mathrm{cm}}^{-1}$ in 21 increments to mimic low and high melanin concentration, and $\mu_{a,sub}$ was varied between 0.01 and $0.75\;{\mathrm{cm}}^{-1}$ in 75 increments to mimic a variety of blood volume and oxygen saturation of hemoglobin values.

For a specific λ, the two-layer LUT could be considered in 3D form with the pair of measured $R_{d}(f_{x})$ and assumed $\mu_{a,epi}$ as inputs and $\mu_{a,sub}$ and $\mu_{s,sub}^{\prime }$ as output parameters. In practice, 3D interpolation was found to be prohibitively slow in MATLAB for pixel-by-pixel analysis. Instead, one-dimensional interpolation (MATLAB’s “interp1”) was performed to obtain tables specific to the optimized $\mu_{a,epi}(\lambda )$. This operation yielded a conventional 2D LUT, where a pair of $R_{d}(f_{x})$ produced a pair of $\mu_{a,sub}$ and $\mu_{s,sub}^{\prime }$ for an optimized $\mu_{a,epi}$ and a constant $\mu_{s,epi}^{\prime }$. The extraction process was repeated for each of the 3 SFDI wavelengths to generate $\mu_{a,sub}(\lambda )$ and $\mu_{s,sub}^{\prime }(\lambda )$.

Additionally, a conventional homogenous SFDI LUT was generated for comparison purposes. Using a homogeneous one-layer geometry, all other properties were maintained, with $\mu_{s}^{\prime }$ ranging from 4 to $20\;{\mathrm{cm}}^{-1}$ in 17 increments and $\mu_{a}$ ranging from 0.01 to $0.75\;{\mathrm{cm}}^{-1}$ in 75 increments.

#### 2.3.3 Optimization of epidermis layer absorption coefficient

The purpose of this optimization procedure is to produce the values of $\mu_{a,epi}(\lambda )$ which, as inputs to the two-layer model, allow for quantification of $\mu_{a,sub}(\lambda )$ and $\mu_{s,sub}^{\prime }(\lambda )$. A distinction must be made between the $\mu_{a,epi}$ used in the two-layer model and the true absorption coefficient of a patient’s epidermis, which will be equal only if the patient’s epidermal thickness is equal to that used in the model (110  μm). Without *a priori* knowledge of the participant-specific epidermal thickness, direct estimation of $\mu_{a,epi}(\lambda )$ is not possible with this method. Instead, we aim to optimize for the product of epidermal thickness and $\mu_{a,epi}$. Because epidermal thickness is much larger than $1/\mu_{s,sub}^{\prime }(\lambda )$, we are assuming the effect of scattering within the epidermis to be small, and optical thickness of the epidermis can be approximated as the product of epidermal thickness and $\mu_{a,epi}$.

Additionally, this optimization procedure does not attempt to fit for $\mu_{a,epi}$ at the three wavelengths independently. It is assumed that melanin is the only contributing absorber in the epidermis and melanin is modeled to follow the extinction coefficient ($\varepsilon_{mel}$) spectrum given as

$$\varepsilon_{mel}=e^{-0.009\lambda +12.93}.$$ (5)

This reduces the problem to a single degree of freedom, in which the quantity to be optimized can be defined as the unitless parameter we term melanin index (MI),

$$\mathrm{MI}=d_{epi}\cdot \mu_{a,epi}(662).$$ (6)

MI is the approximate optical thickness at 662 nm and is proportional to the amount of melanin per unit area of skin.

To determine MI for each human subject volunteer, $\mu_{a,sub}(\lambda )$ was calculated for a portion of the baseline measurement from each trial using a range of $\mu_{a,epi}$ values in the two-layer model. Because specular reflections were present in some measurements, pixels in the demodulated images were identified for which magnitude at $f_{x}=0$, M(0), differed by more than 2.5 standard deviations from the mean value of the image. Pixel locations, (x,y), meeting this rejection criteria at any time point, t, or wavelength, λ, were excluded from the $\mu_{a,epi}$ optimization procedure. An average diffuse reflectance, $R_{d}(f_{x},\lambda )$, was calculated over the remainder of the image and over timepoints between t=10 and t=55  s. This time period comprised the majority of the 60 s baseline of the measurement, excluding the first 10 s when device repositioning occurred in some trials. After determination of average $R_{d}(f_{x},\lambda )$ for each trial, 161 potential values of MI were considered on the range of 0 to 0.176 (inclusive, evenly spaced), from which $\mu_{a,epi}(\lambda )$ at each wavelength was obtained. Given the model layer thickness of 0.011 cm, this range corresponded to values of $\mu_{a,epi}(662)$ between 0 and $16\;{\mathrm{cm}}^{-1}$, from which $\mu_{a,epi}(735)$ and $\mu_{a,epi}(859)$ were calculated based on Eq. (5).

Using the two-layer LUT described in Sec. 2.3.2, subcutaneous optical properties were extracted from $R_{d}(f_{x},\lambda )$ for the above calculated $\mu_{a,epi}(\lambda )$ and the constant $\mu_{s,epi}^{\prime }(\lambda )$, yielding 161 spectra of $\mu_{a,sub}(\lambda )$ and $\mu_{s,sub}^{\prime }(\lambda )$. From $\mu_{a,sub}(\lambda )$, HbO and Hb concentrations were calculated based on least squares regression to the extinction coefficients of hemoglobin.^43^

$$[\begin{array}{c} e_{662}\\ e_{735}\\ e_{859} \end{array}]=[\begin{array}{c} \mu_{a,sub}(662)\\ \mu_{a,sub}(735)\\ \mu_{a,sub}(859) \end{array}]-[\begin{array}{cc} \varepsilon_{HbO,662} & \varepsilon_{Hb,662}\\ \varepsilon_{HbO,735} & \varepsilon_{Hb,735}\\ \varepsilon_{HbO,859} & \varepsilon_{Hb,859} \end{array}]*[\begin{array}{c} C_{\mathrm{HbO}}\\ C_{\mathrm{Hb}} \end{array}],$$ (7)

where molar extinction coefficients are denoted by ε, $C_{\mathrm{HbO}}$ and $C_{\mathrm{Hb}}$ are molar concentration of oxy- and deoxyhemoglobin, respectively, and the vector on the left side of Eq. 7 is the error term to be minimized. Each MI tested yielded a $\mu_{a,sub}(\lambda )$ spectrum, which was converted to $C_{\mathrm{HbO}}$ and $C_{\mathrm{Hb}}$ by minimizing the mean-squared error (MSE) of Eq. (8), defined as

$$\mathrm{MSE}=\frac{1}{3}\cdot ({e_{662}}^{2}+{e_{735}}^{2}+{e_{859}}^{2}).$$ (8)

The optimal value of MI for each subject was determined by minimizing the final MSE of the fit to the hemoglobin extinction coefficient spectra. This was based on the assumption that the lowest error would correspond to the MI value at which epidermal melanin was adequately accounted for and $\mu_{a,sub}(\lambda )$ represented only the effects of subcutaneous hemoglobin absorption.

#### 2.3.4 Software phantom validation and sensitivity analysis

To test this optimization algorithm, several MC forward simulations were performed representing eight skin pigmentation levels, MI=0, 0.005, 0.01, 0.04, 0.07, 0.1, 0.13, and 0.16, with all other simulation parameters held constant. For all, epidermis layer thickness $d_{epi}$ of the simulation input was set to 0.011 cm, and $\mu_{a,epi}(\lambda )$ was calculated from MI based on Eqs. (5) and (6). $\mu_{s,epi}^{\prime }(\lambda )$ was set to 22.16, 16.89, and $11.62\;{\mathrm{cm}}^{-1}$ for 662, 735, and 859 nm (matching the values used in the two-layer LUT), repectively, and $\mu_{s,sub}^{\prime }(\lambda )$ was set to 10.21, 8.94, and $7.78\;{\mathrm{cm}}^{-1}$. $\mu_{a,sub}(\lambda )$ was derived from a tHb concentration of 20  μM and tissue oxygen saturation of 75%, representative of typical values for healthy breast.^2^ As these optical properties are wavelength dependent, three simulations were performed for each MI value, corresponding to the three SFDI wavelegnths. The ability of the optimization method to recover ground truth MI was tested. Subcutaneous optical properties, $\mu_{a,sub}(\lambda )$ and $\mu_{s,sub}^{\prime }(\lambda )$, were calculated using the two-layer model and optimized MI output, and using the homogenous model. In both cases $\mu_{a,sub}(\lambda )$ was converted to tHb and ${\mathrm{StO}}_{2}$. Percent error between the simulation ground truth and tHb and ${\mathrm{StO}}_{2}$ obtained from the layered model was calculated for each MI and compared to the same percent error using the homogeneous model.

In addition to the above simulations, in which $d_{epi}$ and $\mu_{s,epi}^{\prime }(\lambda )$ were kept matched to the values assumed by the two-layer LUT, simulations were also performed to assess the effect of either epidermis thickness or scattering mismatch on the accuracy of subcuaneous property quantification. For the thickness mismatch simulations, $d_{epi}$ was varied in nine increments between 0.003 and 0.019 cm for each MI. $\mu_{a,epi}$ was again obtained from MI and $d_{epi}$, with the effect that $\mu_{a,epi}$ varies to compensate for variations in $d_{epi}$ and produce consistent MI. Simulations were also performed in which scattering amplitude is varied. Beginning with the values matching the LUT, simulations were run with all $\mu_{s,epi}^{\prime }(\lambda )$ uniformly increased by 10%, 20%, and 30%, and decreased by 10%, 20%, and 30%. Finally, simulations were perfomed in which scattering power b is varied. By selecting the middle wavelength (735 nm) as the reference wavelength in Eq. 3, the value of b was to produce new $\mu_{s,epi}^{\prime }(662)$ and $\mu_{s,epi}^{\prime }(859)$. The value of b was increased by 10%, 20%, and 30%, and decreased by 10%, 20%, and 30%. For each of these variations, the resulting error in tHb and ${\mathrm{StO}}_{2}$ quantification is calcualted for the eight MI values.

### 2.4 Processing of Dynamic Breast Compression Data

After optimizing the value of MI for each subject from their baseline measurement, the hemodynamic changes induced by compression of the breast tissue were calculated. This was achieved by calculating $\mu_{a,sub}(x,y,\lambda ,t)$ and $\mu_{s,sub}^{\prime }(x,y,\lambda ,t)$ on a pixel-by-pixel basis. Because of the presence of specular reflections and other artifacts in some images, pixels were eliminated if $\mu_{a,sub}(x,y)$ at any λ or any t differed by more than eight standard deviations from the average over a region ∼20  mm square in the center of the image. HbO(x,y,t) and Hb(x,y,t) were then computed from $\mu_{a,sub}(x,y,\lambda ,t)$ and converted to tHb(x,y,t) and ${\mathrm{StO}}_{2}(x,y,t)$ as described in Sec. 2.1. The circular field of view was partitioned into eight regions of interest (ROIs) and pixel values were averaged within each region to produce tHb(t) and ${\mathrm{StO}}_{2}(t)$ time traces. To assess stability in hemoglobin concentration changes during the baseline period and eliminate those regions with baseline drifts, linear fits were performed to the ${\mathrm{StO}}_{2}(t)$ trace for the time period t=10 to t=55  s. Baseline noise was assessed by calculating the root mean squared deviation of the data from the linear fit. ROIs with baseline slope of magnitude greater than 0.045 percentage points per second or variation >0.4% points were rejected. Linear fits were also performed for the time period t=65 to t=113  s to obtain the rate of change of tissue oxygen saturation during compression.

## 3 Results

### 3.1 Monte Carlo Lookup Tables

**Fig. 2:**
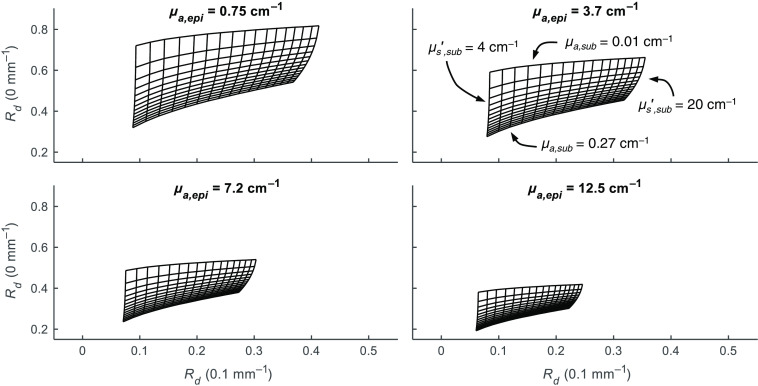
Example of two-layer LUTs with top layer absorption ($\mu_{a,epi}$) fixed at four different values. For all, top-layer scattering is fixed at $16.89\;{\mathrm{cm}}^{-1}$ (corresponding to reduced scatting at 735 nm). Isolines for 14 increments of $\mu_{a,sub}$ are shown from 0.01 to $0.27\;{\mathrm{cm}}^{-1}$. Isolines for $\mu_{s,sub}^{\prime }$ are shown in 17 increments from 4 to $20\;{\mathrm{cm}}^{-1}$.

To demonstrate the effect of the epidermis layer absorption on diffuse reflectance, four Monte Carlo LUTs at varying values of $\mu_{a,epi}$ are shown in Fig. 2. With the same isolines plotted for each, increasing $\mu_{a,epi}$ has the effect of shifting the table to lower values of $R_{d}(0)$ and to a lesser extent lower values in $R_{d}(0.1)$ while compressing the table to a smaller range of $R_{d}$ values in both dimensions. This represents decreased precision in quantifying optical properties at high $\mu_{a,epi}$, as small errors in the $R_{d}$ map lead to larger errors in $\mu_{a,sub}$ and $\mu_{s,sub}^{\prime }$. A given MI and $d_{epi}$ corresponds to a $\mu_{a,epi}$ value for each of the three wavelengths of interest. As determined empirically from *in vivo* data and the optimization procedure described in Sec. 2.3.3, the four LUTs represent a range from low to high melanin concentration in $\mu_{a,epi}(662)$.

### 3.2 Extraction of Melanin Index in Simulated Data

**Fig. 3:**
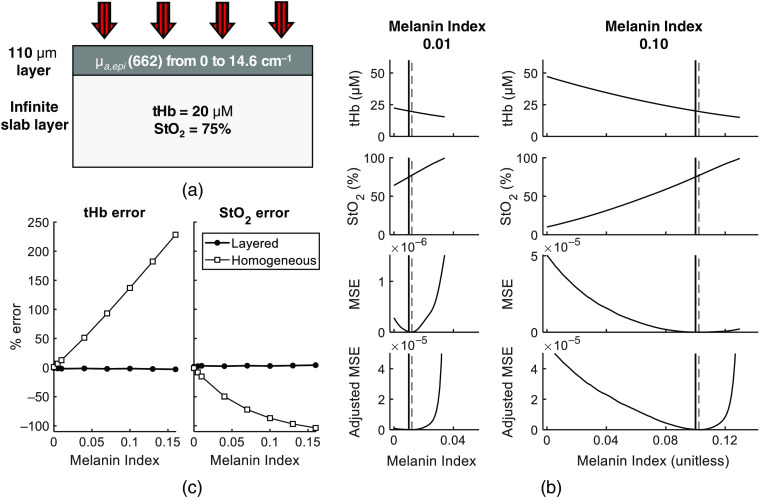
(a) Illustration of the varying absorption properties of the eight multi-wavelength MC forward simulations, having $d_{epi}$ and $\mu_{s,sub}^{\prime }$ matched with the LUT. (b) Demonstration of the optimization of MI for simulated data, showing the effect of assumed MI on recovered tHb and ${\mathrm{StO}}_{2}$ values for two simulations of differing ground truth MI (0.01 and 0.1) and the MSE obtained from fitting the hemoglobin concentrations to $\mu_{a,sub}(\lambda )$. The MI value that produces the lowest MSE is marked with a dotted vertical line and the ground truth MI of the simulation is marked with a solid vertical line. (c) Percent error in tHb and ${\mathrm{StO}}_{2}$ quantification using the layered model (with MI obtained from lowest MSE) compared with a homogeneous model, showing drastically reduced error when the layered model is used.

The forward simulation inputs are summarized in Fig. 3(a). With $d_{epi}$ of 0.011 cm, MI range from 0 to 0.16 corresponds to a $\mu_{a,epi}(662)$ range from 0 to $14.6\;{\mathrm{cm}}^{-1}$. For each MI, analysis with an LUT matching the $\mu_{a,epi}(\lambda )$ corresponding to the ground truth MI was found to reproduce $\mu_{a,sub}$ and $\mu_{s,sub}^{\prime }$ to within 1.4%. To estimate MI based on the optimization procedure in Sec. 2.3.3, we calculated optical properties using LUTs based on a range of MI values. This analysis showed that measurements of $\mu_{a,sub}(\lambda )$, and thus of tHb and ${\mathrm{StO}}_{2}$, were highly sensitive to the assumed value of MI. These trends are shown in Fig. 3(b) for two values of input MI (namely, 0.01 and 0.1.) The solid vertical lines indicate the actual MI value of the forward simulation. The dotted vertical lines correspond to the estimated MI based on minimum MSE obtained when fitting hemoglobin extinction coefficients to measured $\mu_{a,sub}$ for extraction of hemoglobin concentrations.

These results illustrate that the point of minimum MSE slightly over-estimates the ground truth value of MI. This overestimation is reflected in the computation of underlying tHb and ${\mathrm{StO}}_{2}$, illustrated in Fig. 3(c). The mean error of ${\mathrm{StO}}_{2}$ extraction was found to be 2.9%, increasing from 1.6% at MI=0 to 4.2% at MI=0.16. The mean error for tHb was found to be −1.9%, increasing from −0.9% at MI=0 to −3% at MI=0.16. Though error for the layered model is still dependent on MI, it is drastically reduced compared to the error from the homogeneous model, where the error in ${\mathrm{StO}}_{2}$ from MI=0 to MI=0.16 ranges from −0.8% to −103.7%. and error in tHb ranges from 1% to 228.3%. Also plotted in Fig. 3(b) is the adjusted MSE, calculated as $\mathrm{MSE}/(1-{\mathrm{StO}}_{2})$. This adjustment was developed based on the results of the *in vivo* data, detailed below. For simulated data, adjusted MSE yields the same estimate of MI as unadjusted MSE for all cases.

**Fig. 4:**
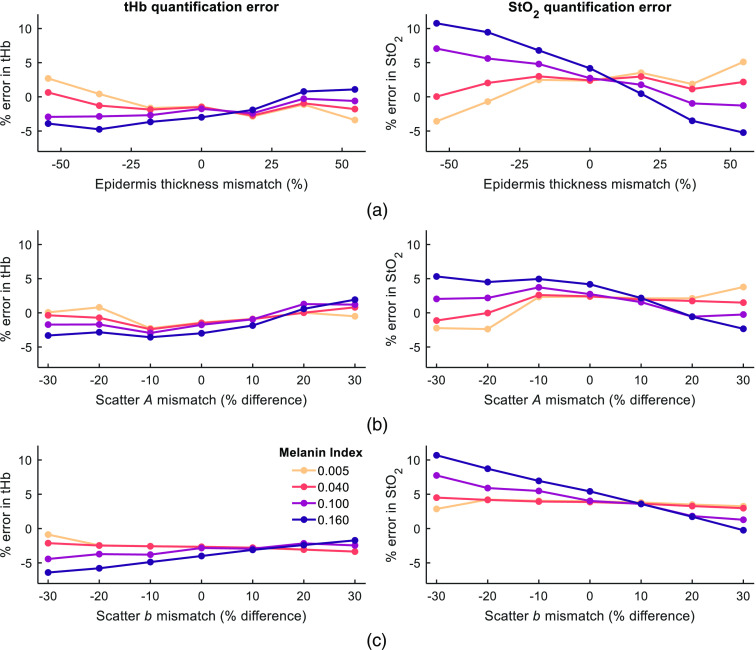
Results of sensitivity analysis showing error in quantification of tHb and ${\mathrm{StO}}_{2}$ for mismatch in (a) epidermis thickness; (b) scattering amplitude; and (c) scattering power for four selected values of MI. For all rows, a negative mismatch represents a value which is lower than that assumed by the model, and a positive mismatch represents a value higher than that assumed by the model.

Error quantification for simulations where $d_{epi}$ and scattering differ from the properties assumed by the two-layer model are summarized in Fig. 4 for MI values up to 0.16. The upper limit of MI in this analysis was determined empirically, exceeding the greatest MI value calculated for the healthy subjects in Sec. 3.3. While a mismatch in scattering properties and layer thickness introduce erroneous results, all errors are lower than 11% and the majority of cases are lower than 5%. Human epidermal thickness is known to vary considerably, with one study reporting a range of ∼50 to 100  μm for skin on breast (female), 90 to 165  μm for skin on abdomen and 100 to 175  μm for skin on the back,^39^ and another reports a range of ∼50 to 90  μm for forearm.^40^ The thicknesses considered for this analysis (between ∼50 and 170  μm) were chosen to cover these ranges for epidermal thickness. These results indicate that though the two-layer model assumes a fixed epidermis thickness and scattering properties, this optimization procedure retains validity in estimating subcutaneous tHb and ${\mathrm{StO}}_{2}$ even as epidermis properties differ from their assumed values.

### 3.3 Extraction of Melanin Index in Human Data

**Fig. 5:**
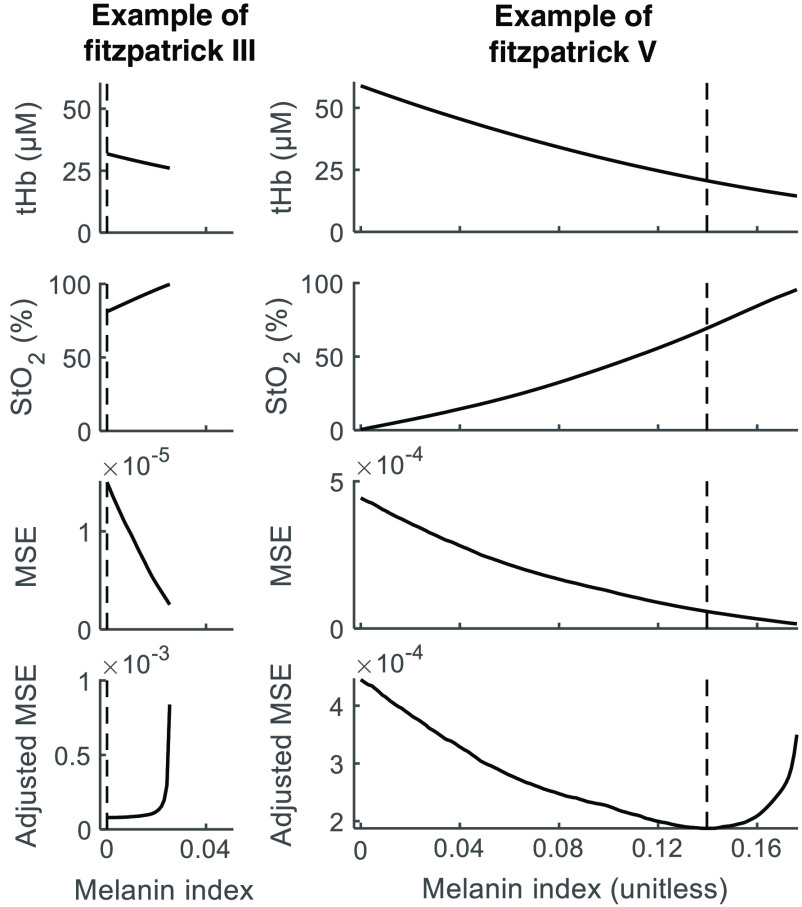
Example of the effect of assumed MI on recovered tHb and ${\mathrm{StO}}_{2}$ values for two subjects of different Fitzpatrick type. The third row shows the effect on MSE obtained from fitting the hemoglobin concentrations to $\mu_{a,sub}(\lambda )$, and the bottom row shows the adjusted MSE, obtained from $\mathrm{MSE}/(1-{\mathrm{StO}}_{2})$. The MI value that produces the lowest adjusted MSE is marked with a dotted vertical line.

The effect of varying assumed MI on the goodness of fit of hemoglobin concentrations to $\mu_{a,sub}(\lambda )$ is examined for *in vivo* breast data. This trend, along with the effect on estimated tHb and ${\mathrm{StO}}_{2}$, is shown in Fig. 5 for two representative measurements from subjects of different Fitzpatrick scores.

Similar to the trend observed in the simulated data, increasing assumed MI produced lower estimates of tHb and higher estimates of ${\mathrm{StO}}_{2}$. Unlike the simulated data, the relationship of MSE to MI was not unimodal but monotonically decreasing for all measurements, exhibiting the lowest value at the point corresponding to ${\mathrm{StO}}_{2}$ of 100%. Values above 100% or below 0% are considered undefined, as they would correspond to negative chromophore concentrations. This may be owing to the shape of the extinction spectrum of deoxyhemoglobin, which exhibits a dip around 740 nm. In all measurements, the measured value of $\mu_{a,sub}(735)$ is greater than that which would be predicted by the calculated hemoglobin concentrations alone. The failure of the measured $\mu_{a,sub}(\lambda )$ to conform to the shape predicted by the extinction spectra of hemoglobin may be due to the presence of other chromophores, such as water and lipid, which are not accounted for in this study. Regardless of its origin, the mismatch between observed and predicted $\mu_{a,sub}(735)$ leads to relatively large MSE when the fit yields physiologically reasonable values of Hb concentration, and much lower MSE for extremely low Hb concentrations, because the spectrum of HbO does not exhibit the dip in this range and a better fit is obtained.

**Fig. 6:**
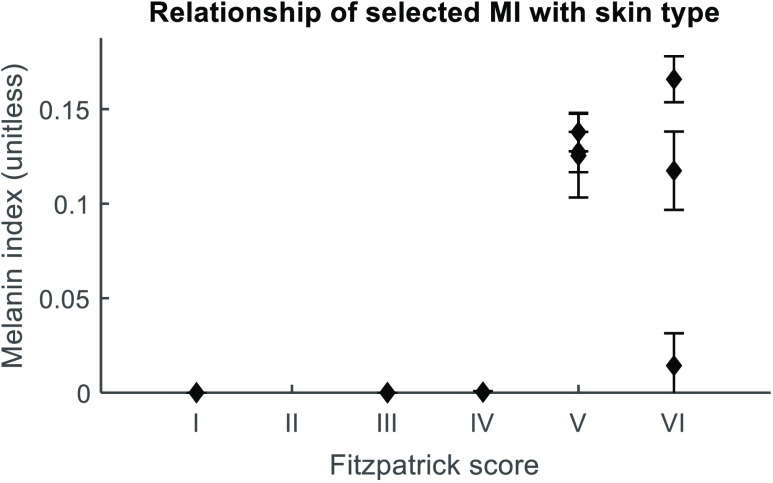
Relationship of Fitzpatrick skin score with MI, where MI is selected to produce the lowest error in fitting hemoglobin concentrations to $\mu_{a,sub}(\lambda )$. Each diamond represents one subject and error bars are standard deviation of the individual measurements. Due to the repeat imaging session by some subjects, n ranges from 4 to 8 measurements. Types I and IV represent one subject each. Types III, V, and VI represent three subjects.

Based on the apparent bias against high values of Hb, an adjusted MSE was introduced, in which MSE is divided by the corresponding percentage of Hb at that MI, ($1-{\mathrm{StO}}_{2}$). When this adjustment is applied, a minimum is present which corresponds to an average ${\mathrm{StO}}_{2}$ value over all measurements of 68±11% (mean±std) and average tHb concentration of 19.2±5  μM. These ranges are physiologically reasonable based on literature for healthy breast.^2^. The MI corresponding to minimum adjusted MSE is identified for each measurement. These values are averaged for each subject, and plotted against subject’s Fitzpatrick skin type in Fig. 6. All subjects of Fitzpatrick score I, II, and III received an optimal MI of 0, the single type IV subject received 0.003, and subjects of Fitzpatrick V and VI had a mean MI of 0.115 with a standard deviation of 0.052 (range: 0.014 to 0.166). Our results indicate that the algorithm is sensitive to higher MI values, as seen for participants of Fitzpatrick types V and VI exhibited higher MI, whereas the algorithm applied to participants of type I, II, III, and IV showed no sensitivity to MI, indicating that a homogenous reconstruction may be applicable.

**Fig. 7:**
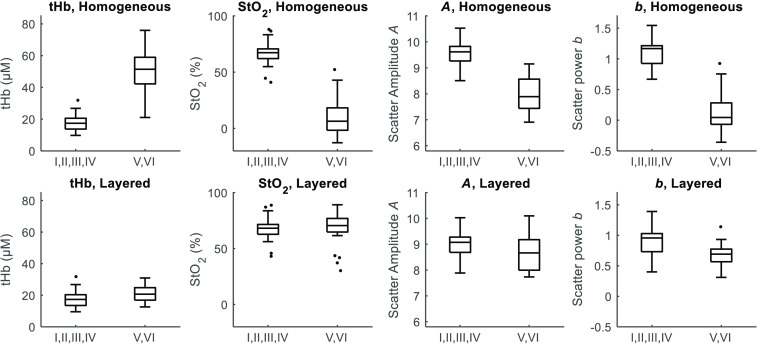
Boxplots of optical properties obtained with homogeneous (top row) and layered (bottom row) inverse models for trials on Fitzpatrick I, II, III, and IV subjects (lighter skin tone) and subjects of Fitzpatrick V and VI (darker skin tone). The layered model result is based on the adjusted MSE described above and does not require *a priori* assumption of absorption coefficient of either layer. Differences between groups in the layered model values were assessed with two-sample t-tests, and a statistically significant difference is found to remain for tHb (p=0.012) and scatter power b (p<0.001). For the homogeneous model values, p<0.001 was found for all four parameters.

Using the adjusted MSE analysis, the optical properties could be extracted for all subjects. Specifically, each subject’s average estimated MI value (obtained from multiple measurements) is used to calculate baseline optical properties for all of that subject’s measurements. Baseline optical properties are also calculated for every measurement using the homogeneous LUT. Box plots of tHb, ${\mathrm{StO}}_{2}$, scatter amplitude A, and scatter power b before and after correction are shown in Fig. 7 for two groupings of Fitzpatrick skin type: I, II, III, and IV (lighter skin tone) and V and VI (darker skin tone).

Optical properties derived from the homogenous model reveal separation between groups not only for tHb and tissue saturation but also for scattering power and amplitude. Adjusted MSE-based correction brings the two groups closer together for each property considered. For the layered model results, the mean and standard deviation of tHb concentration was found to be 17.7±4.6  μM for Fitzpatrick I, II, III, IV, and 21±5  μM for Fitzpatrick V,VI, both in line with that reported by Grosenick et al.,^2^ ranging from 12.6 to 19.2  μM tHb for healthy breast.^2^ Similarly, using the layered model, ${\mathrm{StO}}_{2}$ for the two groups was 67.9±9.3% and 68±13.6%, respectively, comparable to values reported by Grosenick et al.^2^ of 67.7% to 74%. Layered model-derived scatter power, b, for the two groups was 0.90±0.24 and 0.67±0.18 compared to Grosenick et al. reporting values from 0.58 to 0.99. We also evaluated whether age is a contributing factor to the extracted optical data. Our data suggest (results not shown here) that there is a greater spread of optical properties with age when using the homogenous model. The greater difference in the homogenous assumption is likely explained due to higher average age of the Fitzpatrick V and VI subjects (48 years) than the Fitzpatrick I, II, III, and VI subjects (34 years). After correction with the layered model, no differences in optical properties were found with age.

### 3.4 Breast Compression Dynamic Results

**Fig. 8:**
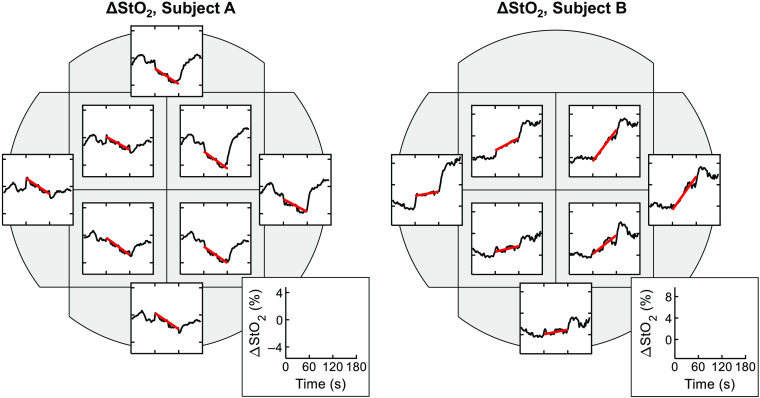
Change in ${\mathrm{StO}}_{2}$ before during and after compression for two measurements taken from different subjects on different regions of the breast. The eight ROIs of each measurement are illustrated as gray shaded areas with an axis overlaid plotting the average change from baseline of ${\mathrm{StO}}_{2}$ in that region (black line), with a linear fit during the compression period overlaid in red. The missing axis represents a region rejected for unstable baseline or artifacts. Inset: x and y axis scale, which is consistent within each subject. The y axis has units of $\Delta {\mathrm{StO}}_{2}$ (%), and x axis has units of seconds. Tick marks at 60 and 120 s mark the beginning and end of the compression period.

The tHb response to localized tissue compression with our handheld SFDI device was found to be consistent with tissue blanching and reperfusion, with tHb decreasing upon initiation of compression onset and recovering quickly after compression release. The response in ${\mathrm{StO}}_{2}$ was more variable and was therefore analyzed spatially according to eight ROIs as shown in Fig. 8. Out of 568 regions available for analysis, 132 of these were rejected due to lack of stable ${\mathrm{StO}}_{2}$ baseline (n=70) or for optical artifacts (n=63) such as specular reflection encompassing more than 60% of that region’s pixels. In the remaining 435 regions, a trend toward decreasing ${\mathrm{StO}}_{2}$ was observed during compression but with considerable variation. While the trend in ${\mathrm{StO}}_{2}$ sometimes differs within different regions of one measurement, inter-measurement and inter-subject variability was found to be even greater. Two representative measurements are shown in Fig. 8, one representing primarily decreasing ${\mathrm{StO}}_{2}$ trends in all eight regions and one representing primarily increasing ${\mathrm{StO}}_{2}$ trends. Plots of ${\mathrm{StO}}_{2}$ response to compression for all measurements are available in Figs. S1–S19 in the Supplementary Material. The red lines in Fig. 8 show a linear fit to the ${\mathrm{StO}}_{2}$ time trace during compression.

**Fig. 9:**
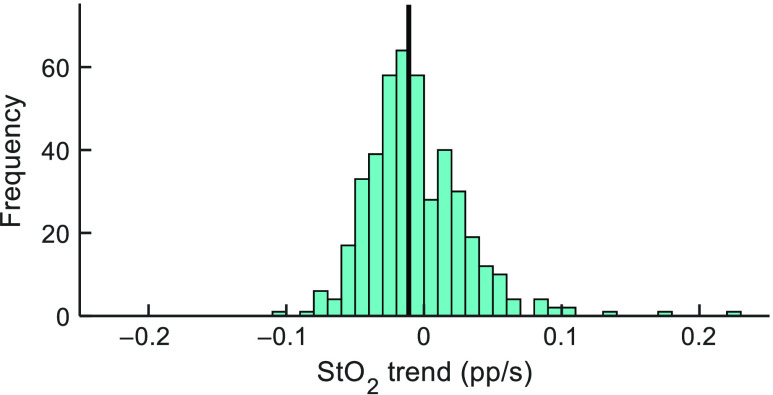
Histogram of distribution of ${\mathrm{StO}}_{2}$ slopes during compression for 470 ROIs. ${\mathrm{StO}}_{2}$ slope is expressed in units of percentage points per second (pp/s). The median slope (marked with black vertical line) occurs at −0.011  pp/s.

The overall variability of the slope of the ${\mathrm{StO}}_{2}$ responses (pp/s) across all ROIs is represented by the histogram in Fig. 9.

**Fig. 10:**
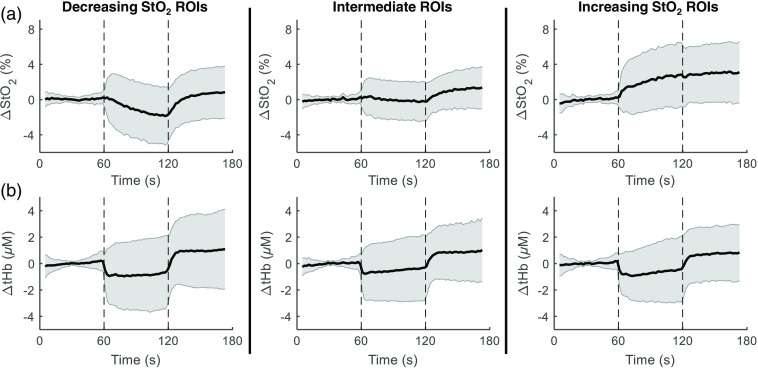
Illustration of average change from baseline of (a) ${\mathrm{StO}}_{2}$ and (b) tHb for ROIs grouped according to ${\mathrm{StO}}_{2}$ slope during compression. Shaded region indicates standard deviation. From left to right, n=145, 145, and 145 ROIs. tHb exhibits a consistent U-shaped response across all three groups.

To illustrate the range of ${\mathrm{StO}}_{2}$ responses, the ROIs were segregated into three groups: those with a pronounced decreasing trend, those of intermediate slope, and those of pronounced increasing trend. The average responses of these three groups, shown in Fig. 10, indicate that the ${\mathrm{StO}}_{2}$ responses can be quite variable between different regions and across subjects, whereas the tHb response shows a more consistent decrease during compression. The group averages correspond to n=145, 145, and 145 ROI averaged time traces.

The ROIs were further analyzed in terms of the Fitzpatrick score of the subject they were obtained from. Again splitting the group into Fitzpatrick scale I, II, III, IV and V and VI, we found the ROIs trending down in ${\mathrm{StO}}_{2}$ contain 118 from I, II, III, IV, and 27 from V and VI, the ROIs exhibiting little change in ${\mathrm{StO}}_{2}$ contain 106 and 39, respectively, and the ROIs exhibiting increasing ${\mathrm{StO}}_{2}$ contain 66 and 79. While the distribution is not equal in all trends, there is not a clear distinction between trends and Fitzpatrick scale.

## 4 Discussion

Here, we presented the application of a two-layer LUT inverse model to measurements of hemodynamics in the healthy breast, using a handheld SFDI instrument. The value of MI (approximate optical thickness at 662 nm) was estimated by an optimization procedure based on examining the fitting error obtained when calculating hemoglobin concentrations from measured optical properties. This model requires the assumption of the thickness of the epidermis layer, however we demonstrate reasonable accuracy in quantifying subcutaneous properties in simulations with epidermal thickness differing significantly from the model assumption. This is the case because diffuse reflectance was primarily influenced by the product of thickness and $\mu_{a,epi}$, with only minor independent contributions. Thus, the assumption of an epidermal thickness does not substantially impede the recovery of subcutaneous properties because $\mu_{a,epi}$ is allowed to vary to compensate. This is consistent with Yudovsky et al.^33^ who reported significant coupling between surface layer thickness and surface-layer absorption coefficient. Human epidermal thickness is known to vary widely, and the 110-μm thickness chosen for this study is with the range of reported thicknesses.^33^^,^^39^^,^^40^

An additional weakness of this model is the simplicity of the two-layer design. The bulk layer was termed “subcutaneous” though it includes both dermal and subcutaneous tissues. When used to measure breast tumors, compression can be used to reduce the depth of the lesion from the surface, but significant partial volume effects will still be present from healthy tissue covering the tumor, including the microvasculature of the dermis.

In simulated data for which the ground truth bulk-layer absorption is derived from only two chromophores, the error in fitting simulated $\mu_{a,sub}(\lambda )$ to concentrations of those two chromophores can be used to extract $\mu_{a}$ of the surface layer (and thus tHb and ${\mathrm{StO}}_{2}$) with a small error around 2% to 3%. In human data, which suffers from noise and the presence of other chromophores in tissue, this method cannot be applied in the same manner, due to an apparent bias toward very low deoxyhemoglobin concentration. The adjustment used in this study, dividing MSE by ($1-{\mathrm{StO}}_{2}$), partially compensated for this error and produced tissue optical properties within the physiological range. However, this was an ad-hoc solution that does not consider the exact nature of the mismatch, and therefore requires future work. Three is the lowest number of wavelengths for which goodness of fit can be assessed when fitting for two chromophores, as it is only one greater than the minimum needed to calculate two concentrations. It is therefore possible that a fourth wavelength could improve the accuracy, with minimal adverse effect on the sampling speed advantages of this system. Additional improvements worthy of consideration are increasing the light intensity or employing a more sensitive detector (especially at 859 nm). This is especially important for subjects with darker skin, as higher values of $\mu_{a,epi}$ are known to make the LUT more sensitive to noise.

When MI was optimized by this method, higher Fitzpatrick scores resulted in higher estimates of MI. All subjects Fitzpatrick IV and lower received an optimal MI value of 0 or near-zero, indicating the absence of any light-absorbing pigment in the epidermis, which is unrealistic. Our results indicate that the method has low sensitivity to low levels of absorption in the epidermis. However, all participants in these group exhibited physiologically reasonable baseline values of tHb and ${\mathrm{StO}}_{2}$ using either the homogeneous model or layered model with MI=0, indicating that the error introduced by a homogeneous inverse model is not significant when melanin concentration is low. Yudovsky et al.^33^ reported a product of epidermal thickness and epidermal absorption coefficient at 660 nm to be ∼0.01 for a Fitzpatrick I subject and 0.06 for a Fitzpatrick IV (no data available on V and VI). A value of 0.06 is higher than that of the lowest MI found in the high Fitzpatrick group in this study (MI=0.014) but lower than the remaining subjects in the high Fitzpatrick group (0.12 to 0.17). That high variation was present in the three Fitzpatrick VI participants may be partially attributed to an imperfect assessment of Fitzpatrick types, which is highly subjective. For example, any subject who reported she experienced neither sunburn nor a noticeable darkening of skin tone in exposed areas after sun exposure was deemed to be a type VI, but we observed a visual difference in skin tone between these participants. Assessment of the Fitzpatrick score alone is thus not a quantitative method for estimating MI due to the significant variation that can occur within type.

While some measurements exhibited a steadily decreasing trend in ${\mathrm{StO}}_{2}$ similar to that observed by Carp et al.^20^ for whole breast compression, other measurements exhibited an increasing trend in ${\mathrm{StO}}_{2}$ often accompanied by a sharp rise in ${\mathrm{StO}}_{2}$ at the onset of compression. The physiological origin of this trend is not clear. It is likely that the location of local compression relative to the location of large blood vessels plays a role in the hemodynamic response. For use in longitudinal monitoring of a breast tumor, it is imperative that the measurement location be kept consistent for this reason. Additionally, a limitation of the current handheld breast imager is that only the average force across the four load cells is recorded, allowing the possibility of uneven application of force by the operator, which may explain the presence of diverging trends in ${\mathrm{StO}}_{2}$ within some images. In the future, better control over the angle of application of force is needed.

## 5 Conclusion

The two-layer inverse model and inexpensive SFDI setup reported here show potential for imaging hemodynamic responses for the prediction of NAC in patients of a variety of skin tones. More work is needed to further validate the estimation of epidermal absorption using our introduced method of optimization based on hemoglobin fitting error.

## Code Data and Materials Availability

Data and code used to generate results and figures are available on Code Ocean, https://doi.org/10.24433/CO.6058805.v1.
