# Supplementary material for: Two-layer spatial frequency domain imaging of compression-induced hemodynamic changes in breast tissue
Source: J Biomed Opt. 2021 May 24;26(5):056005. doi: 10.1117/1.JBO.26.5.056005 (PMC8145994; doi:10.1117/1.JBO.26.5.056005)
Supplement: Supplementary file 1 [file JBO_026_056005_SD001.pdf]

Supplementary Material

Change from baseline of StO_2 in response to local breast compression is plotted below for the 19 imaging sessions. As in Fig. 8, the field of view is divided into eight regions of interest (gray shaded areas) and average ΔStO_2 is calculated for each. A linear fit to the StO_2 response during the compression period is overlaid in red to highlight increasing or decreasing trends. The four measurements of each imaging session correspond approximately to the locations marked on Fig. 1A. Scale of the *y*-axis differs between imaging sessions and is given by the inset example axes. Absent axes indicate an ROI that was rejected for lack of stable baseline, noisy baseline, or presence of specular reflection artifacts. For some imaging sessions, an entire measurement is missing due to device malfunction. In these cases, the shaded regions and heading are omitted.

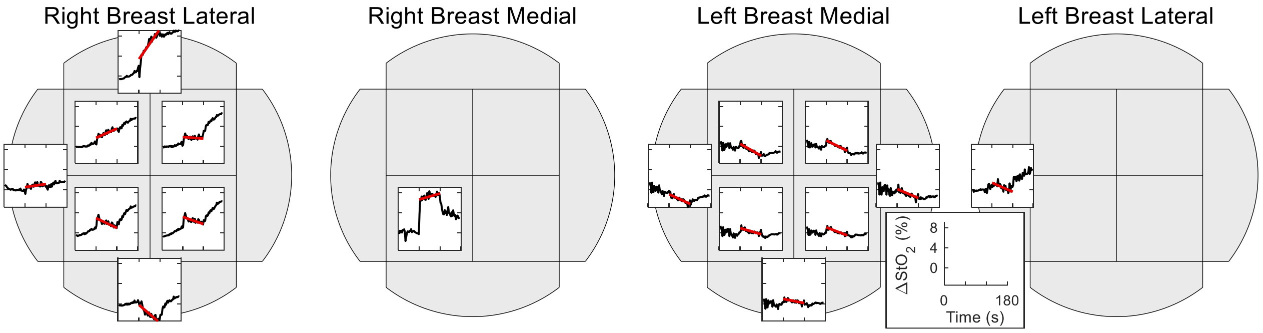

Fig. S1 Change in StO_2 before during and after compression for subject 1, session A.

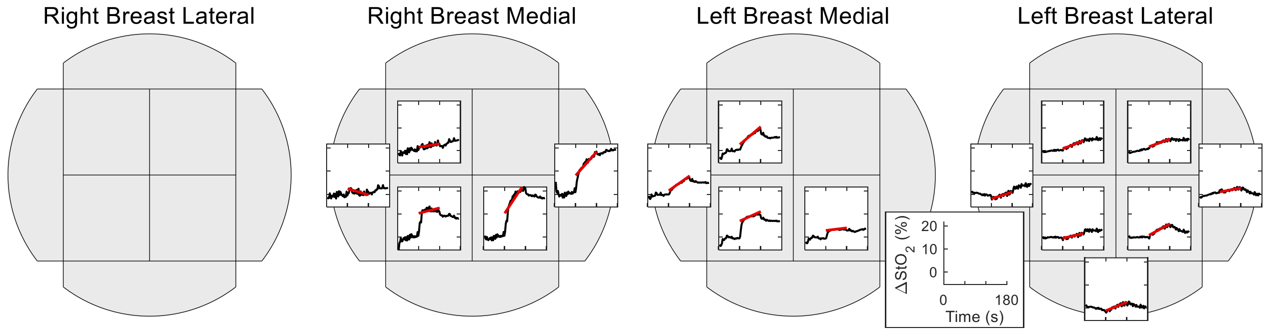

Fig. S2 Change in StO_2 before during and after compression for subject 1, session B.

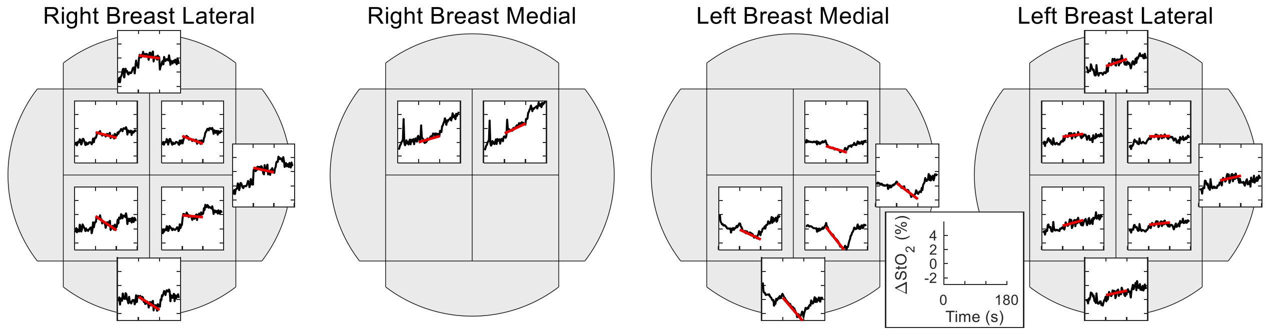

Fig. S3 Change in StO_2 before during and after compression for subject 2, session A.

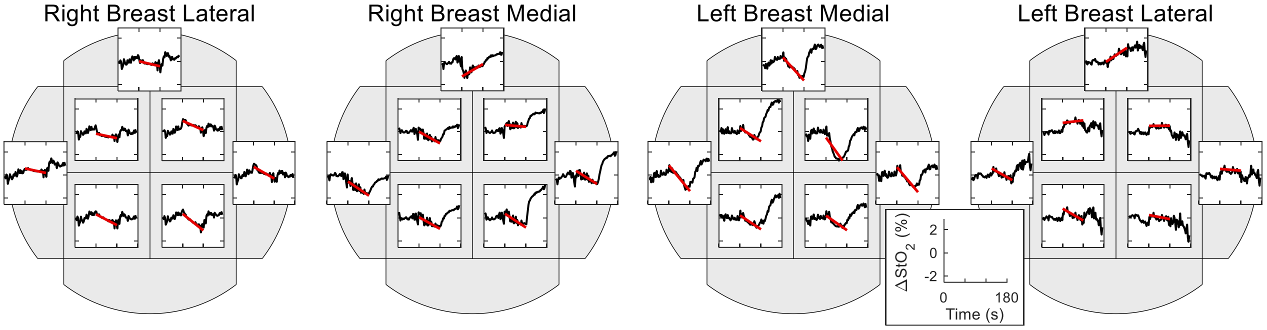

Fig. S4 Change in StO_2 before during and after compression for subject 2, session B.

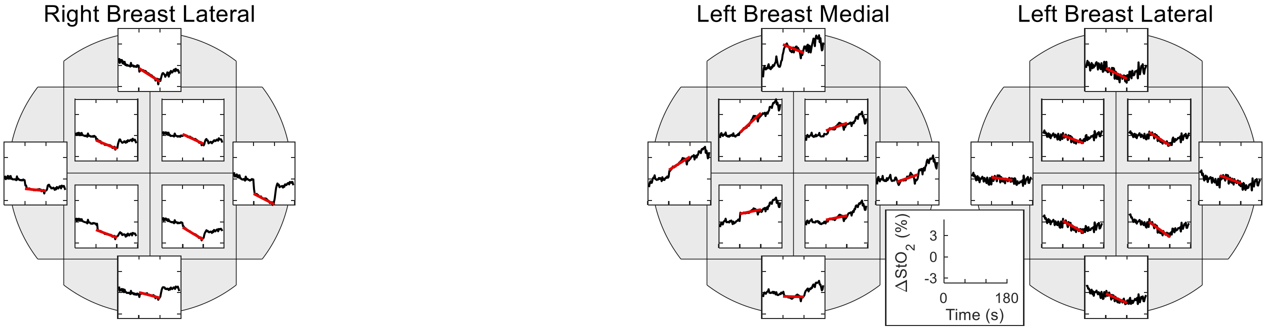

Fig. S5 Change in StO_2 before during and after compression for subject 3, session A.

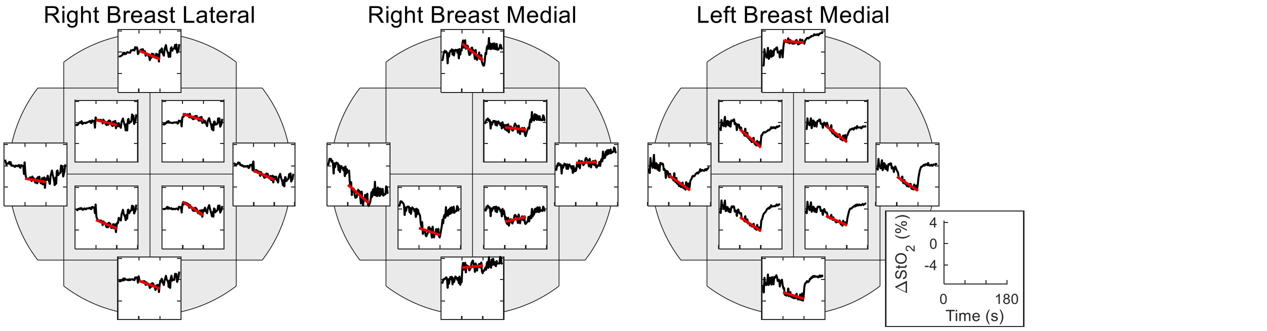

Fig. S6 Change in StO_2 before during and after compression for subject 3, session B.

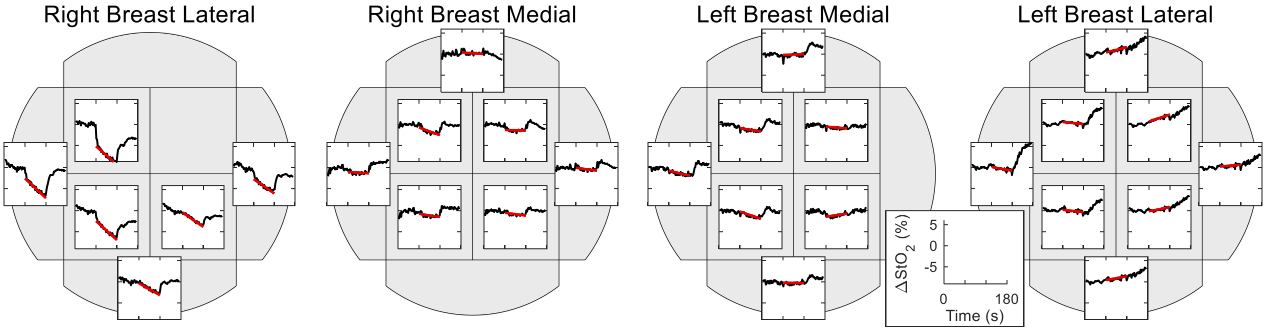

Fig. S7 Change in StO_2 before during and after compression for subject 4.

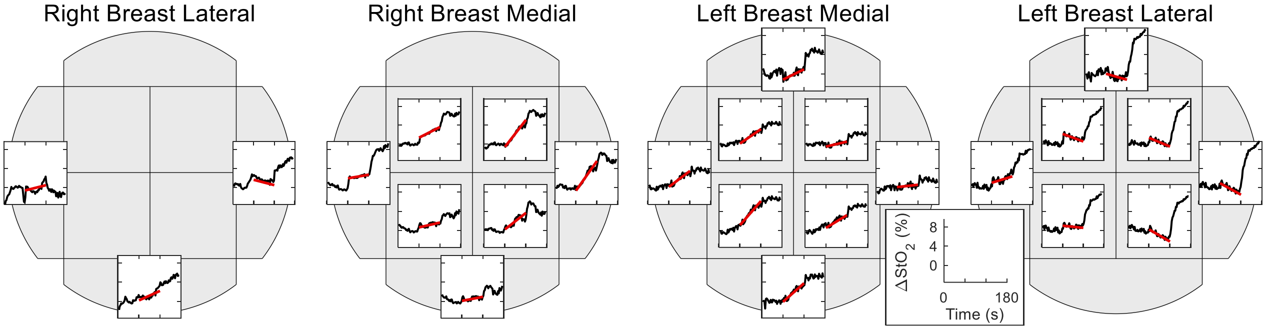

Fig. S8 Change in StO_2 before during and after compression for subject 5, session A.

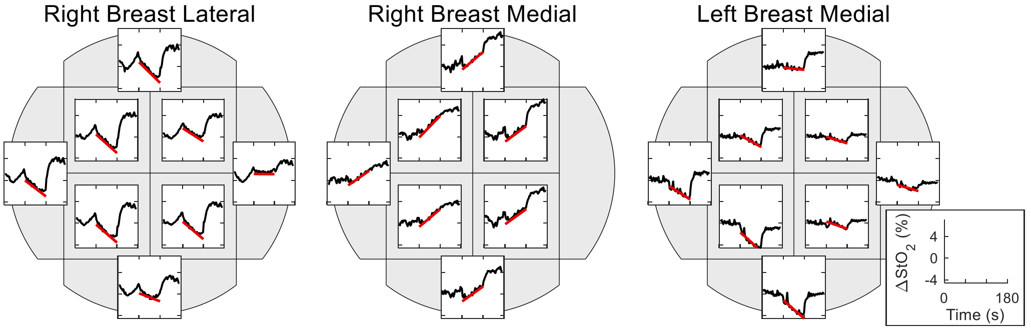

Fig. S9 Change in StO_2 before during and after compression for subject 5, session B.

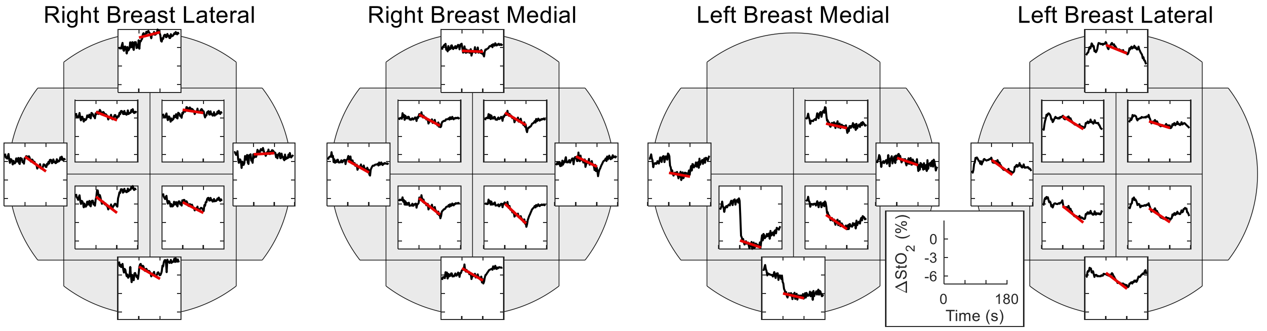

Fig. S10 Change in StO_2 before during and after compression for subject 6, session A.

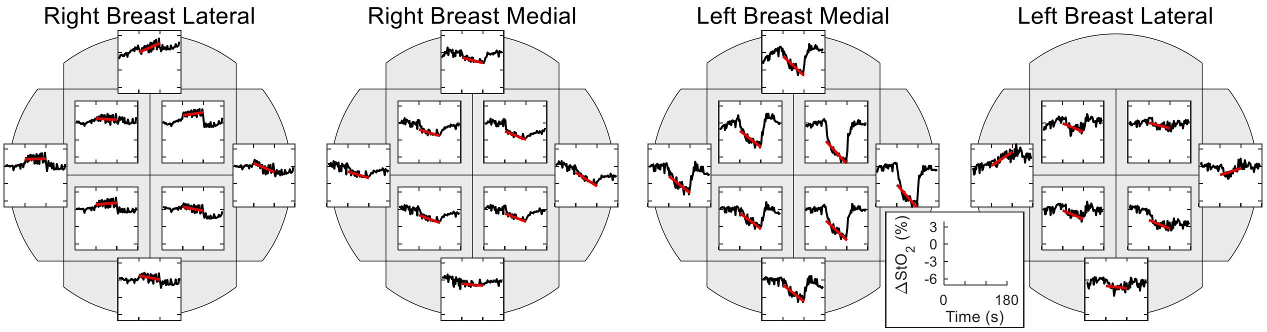

Fig. S11 Change in StO_2 before during and after compression for subject 6, session B.

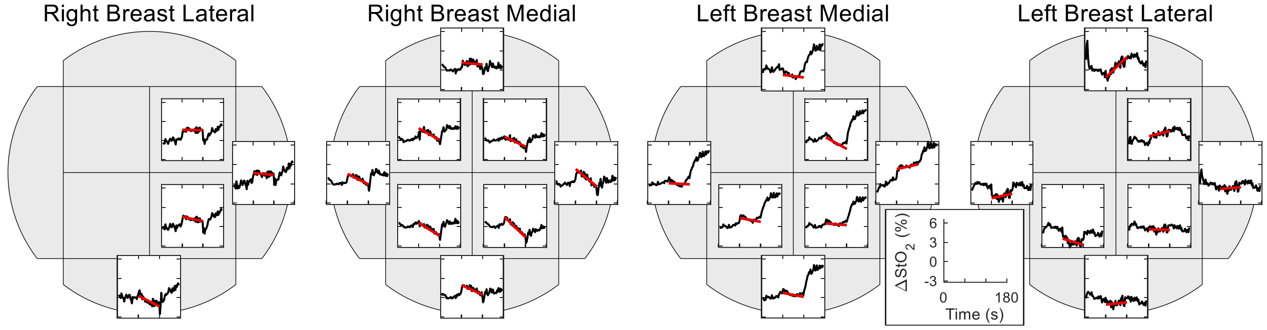

Fig. S12 Change in StO_2 before during and after compression for subject 7.

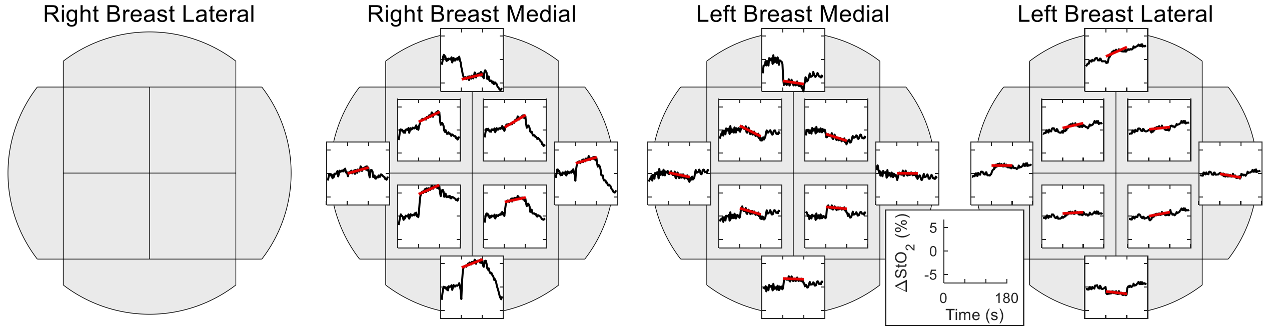

Fig. S13 Change in StO_2 before during and after compression for subject 8, session A.

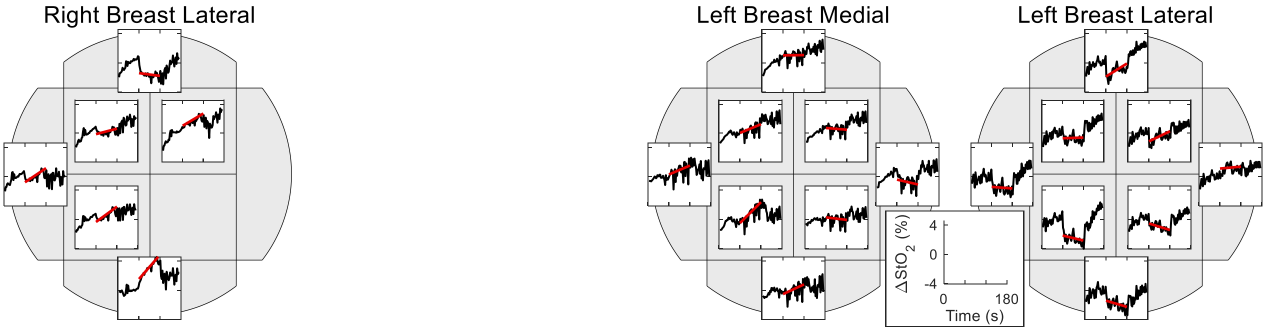

Fig. S14 Change in StO_2 before during and after compression for subject 8, session B.

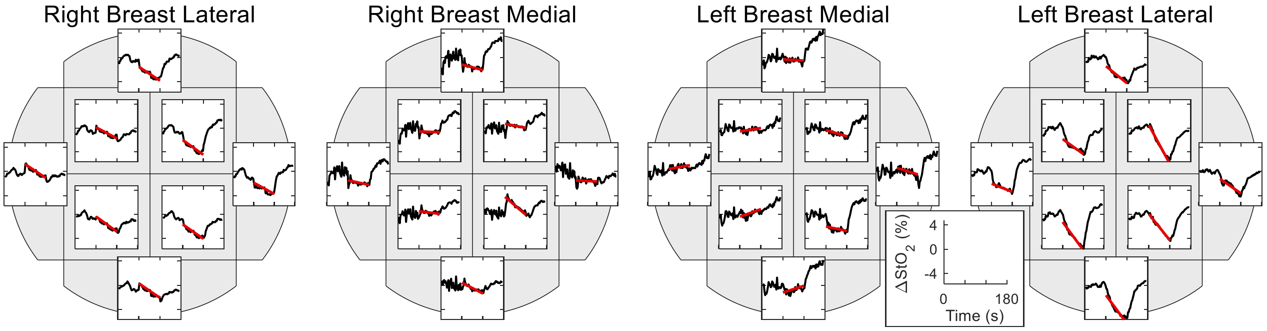

Fig. S15 Change in StO_2 before during and after compression for subject 9.

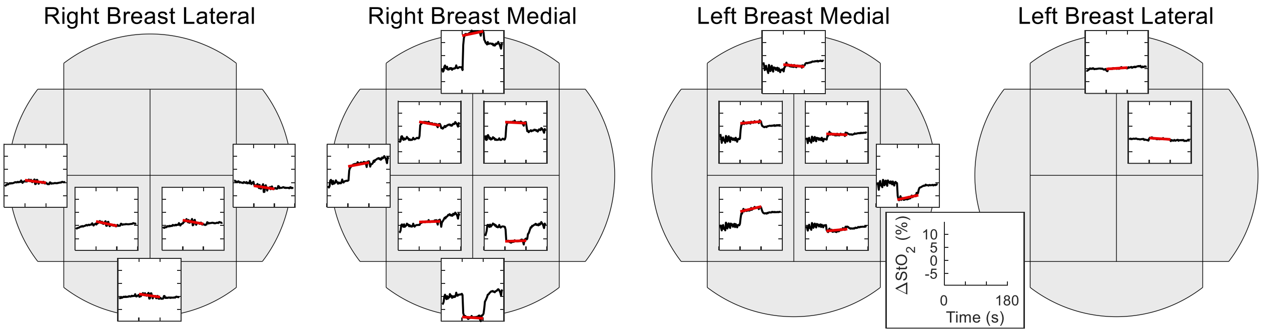

Fig. S16 Change in StO_2 before during and after compression for subject 10.

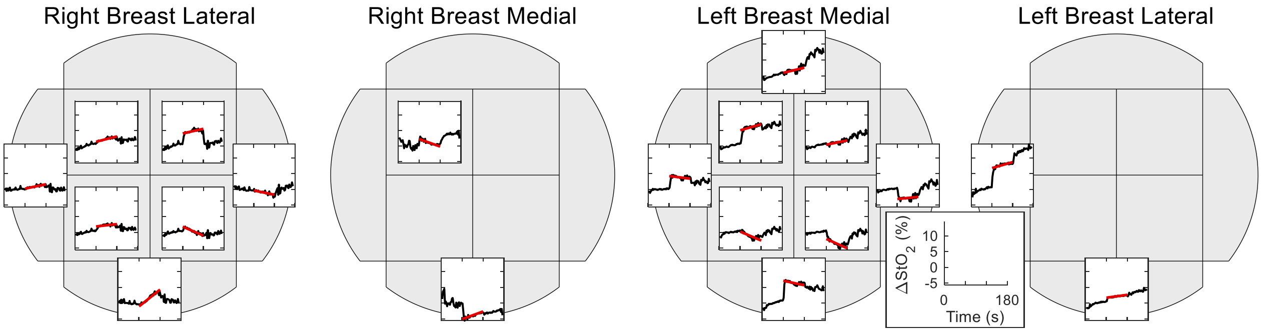

Fig. S17 Change in StO_2 before during and after compression for subject 11.

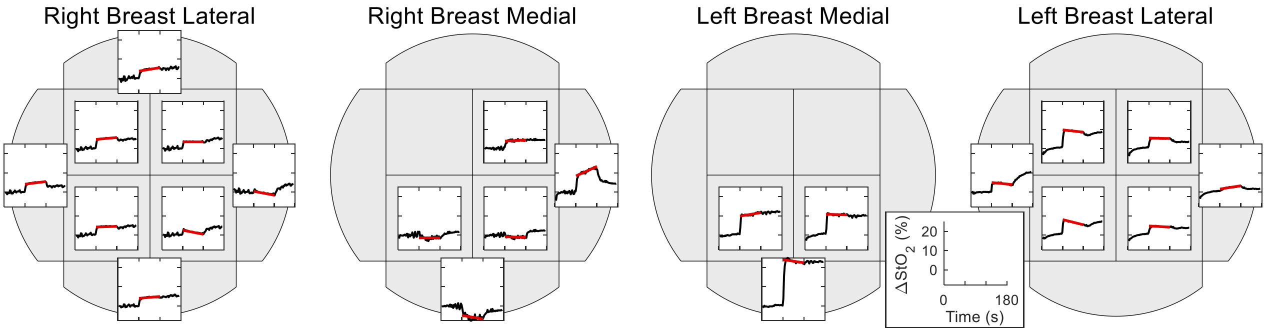

Fig. S18 Change in StO_2 before during and after compression for subject 12.

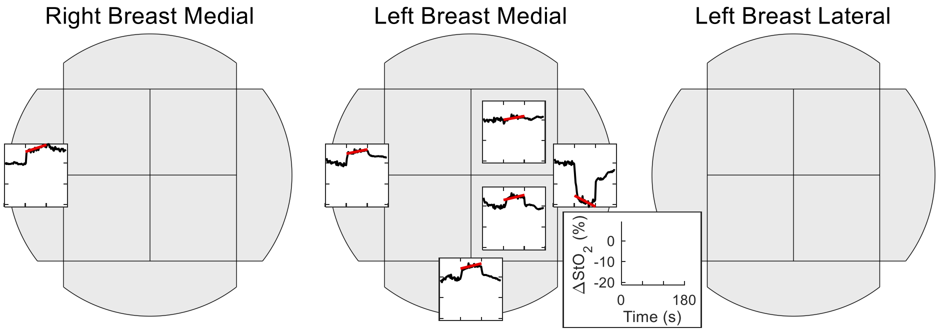

Fig. S19 Change in StO_2 before during and after compression for subject 13.
